# The correlation of crystalline and elemental composition of urinary stones with a history of bacterial infections: TXRF, XRPD and PCR-DGGE studies

**DOI:** 10.1007/s00249-018-1338-7

**Published:** 2018-11-27

**Authors:** Michał Arabski, Ilona Stabrawa, Aldona Kubala-Kukuś, Katarzyna Gałczyńska, Dariusz Banaś, Łukasz Piskorz, Ewa Forma, Magdalena Bryś, Waldemar Różański, Marek Lipiński

**Affiliations:** 1 Department of Biochemistry and Genetics, Institute of Biology, Jan Kochanowski University, Świętokrzyska St. 15, 25-406 Kielce, Poland; 2 Institute of Physics, Jan Kochanowski University, Świętokrzyska St. 15, 25-406 Kielce, Poland; 3 Holycross Cancer Center, Artwińskiego St. 3, 25-734 Kielce, Poland; 4 St. John’s God Hospital, Kosynierów Gdyńskich St. 61, 93-357 Łódź, Poland; 5 Department of Cytobiochemistry, Faculty of Biology and Environmental Protection, University of Lodz, Pomorska St. 141/143, 90-236 Łódź, Poland; 6 2nd Department of Urology, Medical University of Lodz, Pabianicka St. 62, 93-513 Łódź, Poland

**Keywords:** TXRF, XPRD, PCR-DGGE, Urinary stones, *Escherichia coli*, *Proteus* spp.

## Abstract

The aim of this study was to analyze the correlation between past bacterial infections and the type and chemical composition of urinary stones experienced by human patients. Bacteria have been recognized to contribute to urinary stones; however, the role of uropathogens in the development of specific stones has not been extensively investigated. The detection of past bacterial infection (eleven different bacterial species) in urinary stones from 83 patients was made on a DNA level using polymerase chain reaction (PCR) and denaturing gradient gel electrophoresis (DGGE) and correlated with the chemical composition of urinary stones measured using X-ray powder diffraction (XPRD) technique and their elemental composition by total reflection X-ray fluorescence (TXRF). In this study, two scenarios of urinary stones formation mediated by *Proteus* sp. or *Escherichia coli* are presented. The first one is associated with *Proteus* spp. which dominated in 84% of infectious urinary stones and is strongly correlated with struvite and calcium phosphate, in whose matrix additionally strontium, phosphorus, potassium, nickel and zinc are detected. The formation of these stones is closely correlated with urease activity. The second scenario for urinary stone mineralization is associated with *E. coli* identified in weddellite stones, in which matrix iron was detected. In conclusion, the statistical correlations of bacterial infections with crystalline and elemental composition showed that in mixed bacterial infections, one scenario dominated and excluded the second one.

**Electronic supplementary material:**

The online version of this article (10.1007/s00249-018-1338-7) contains supplementary material, which is available to authorized users.

## Introduction

Urolithiasis and urinary tract infections are two urological diseases that may occur independently and simultaneously in the same patient. Urolithiasis occurs in about 10–12% of men and about 6% of women (Asplin et al. 2002; Desai et al. 2017). Urinary tract infections and stones may occur in patients with gout, urinary outflow obstruction and neurogenic bladder (Nseyo and Santiago-Lastra 2017). Additionally, a risk of urinary tract colonization by pathogens is higher in patients who have assumed all sorts of urinary catheters/stents (Klis et al. 2009; Chenoweth and Saint 2016). The possibility of the occurrence and interaction of infection and urolithiasis is common and that is why we tried to assess the frequency and type of pathogens which come into contact with the stone during its presence in the urinary tract.

The use of molecular techniques, based on polymerase chain reaction (PCR) and amplification of 16S rDNA is a rapid and reliable way to identify microorganisms. The combination of PCR and denaturing gradient gel electrophoresis (DGGE) enables the identification of bacterial DNA in various types of urinary stones. DGGE is a sensitive tool for profiling complex microbial populations and direct comparisons can be made between samples run on the same gels. Moreover, DGGE over other profiling techniques gives the possibility of excising bands from the gel for amplification and sequencing (Davies et al. 2004; Tabit 2016). We have made attempts to identify the microorganisms colonizing the urinary tract in different periods of time during the development of urinary stones. On the basis of our previously published data (Kubala-Kukuś et al. 2017) in this study, we statistically analyzed correlations between the chemical composition of urinary stones measured using X-ray powder diffraction (XPRD) technique and their elemental composition determined by total reflection X-ray fluorescence (TXRF) with a history of bacterial infections. We decided to use PCR-DGGE techniques to detect bacterial species at the DNA level. In doing this we took into account the long time period for urinary stone formation and the importance of detecting only bacterial species in urine stone matrix, and not due to any current infection.

## Methods

### Materials and samples’ preparation procedures

Urinary stones were obtained during therapeutic lithotomy with percutaneous nephrolithotomy (PCNL) or ureterorenoscopy (URS) from 83 patients (36 woman and 44 men) of Second Department of Urology, Medical University of Lodz, Poland. The XRPD measurements do not need an additional sample preparation procedure. In the case of TXRF analysis, each sample of human urinary stone was prepared according to the procedure presented elsewhere (Kubala-Kukuś et al. 2017). For genetic studies, DNA was extracted using a modified Swidsinki et al.’s method (Swidsinski et al. 1995). Stone samples of approximately 200 mg were crushed in a 1.5 ml Eppendorf tube and incubated with 600 µl of 1% sodium dodecyl sulfate rotating overnight at room temperature. Lithium chloride solution (7 mol/l) was added to a final concentration of 1.5 mol/l, for precipitating the interfering substances. The DNA was then extracted using QIAamp DNA Investigator Kit (QIAGENE, Hilden, Germany) according to the procedures provided by the manufacturer. For PCR-DGGE studies, the following bacteria were used as controls: *Escherichia coli* ATCC25922, *Klebsiella pneumoniae* ATCC13883, *Morganella morganii* ATCC25830, *Proteus mirabilis* ATCC10005, *Proteus vulgaris* ATCC6896, *Providencia rettgeri* ATCC9250, *Providencia stuartii* ATCC29914, *Pseudomonas aeruginosa* ATCC25668, *Serratia marcescens* ATCC14764, *Staphylococcus aureus* ATCC25923 and *Streptococcus pyogenes* ATCC19615.

### Determination of the crystalline composition of urinary stones by XRPD technique

X-ray powder diffraction measurements were performed in the Bragg–Brentano geometry using X’Pert Pro MPD diffractometer (PANalytical). This diffractometer is equipped with Cu-anode 1.8 kW X-ray tube with linear exit window and PW3050/60 goniometer with an angular resolution of 0.001°. For X-rays diffracted on an analyzed sample, the position sensitive silicon strip detector (X’Celerator) with the crystal dimensions 15 × 9 mm^2^ and 128 strips was used. The detector speeds up the data collection by measuring simultaneously about 2° of 2*θ*. The measurements were performed in the 2*θ* angular range from 5° to 70°. Typical measurement time of one full angular scan was about 30 min. Obtained diffractograms were analyzed qualitatively with Highscore 3.0e program using PDF-2 Release 2009 database of International Centre for Diffraction Data. The detailed information about XPRD methodology is presented elsewhere (Kubala-Kukuś et al. 2017).

### Determination of the elemental composition of urinary stones by TXRF technique

Total reflection X-ray fluorescence measurements were performed with the S2 Picofox spectrometer (Bruker). The characteristic X-rays were excited in the samples with the 30 W Mo-anode X-ray tube operated at 50 kV with an electron current of 0.6 mA. The primary X-ray beam from the tube, monochromated using the Ni/C multilayer monochromator to 17.5 keV energy, was directed onto the studied sample below the critical angle. Fluorescence X-rays from the samples were detected by Peltier-cooled XFlash silicon drift detector having an energy resolution about 160 eV for the Mn–Kα line. The measurements were performed in air. The Picofox spectrometer allows measurement of characteristic X-ray of elements from Mg to U (with exception of Zr to Tc). With spectrometer software (SPECTRA 7), both qualitative analysis of the spectrum and the quantitative analysis of the sample content can be performed (Kubala-Kukuś et al. 2017). The validation of the TXRF technique was carried out by us before actual measurements by analyzing certified water solutions from Merck. The achieved accuracy is on the level of 5–10%.

### Detection of the bacteria in urinary stones by PCR and DGGE

The eubacterial primers (5′-ACTCCTACGGGAGGCAGCAG-3′, 5′-GTATTACCGCGGCTGCTGGCAC-3′) were used in amplification of all ribosomal eubacterial DNA. A 40-bp GC clamp was attached to the reverse primer to obtain PCR fragments suitable for DGGE analysis. A reaction mixture containing approximately 50 ng of template DNA, PCR buffer (10 mM Tris–HCl, pH 8.3; 50 mM KCl; 2.5 mM MgCl_2_; 0.001% gelatin), a 0.2 mM concentration of each PCR primer, a 0.2 mM concentration of each deoxynucleoside triphosphate in a total volume of 50 μl was prepared. The samples were first incubated for 5 min at 94 °C to denature the template DNA and subsequently cooled to 80 °C, at which point 2.5 U of *Taq* DNA polymerase (Applied Biosystems Inc., Foster City, USA) was added. The amplification program was 94 °C for 2 min; 35 cycles of 94 °C for 30 s, annealing for 1 min at 55 °C, and finally 68 °C for 7 min. PCR 16S rDNA fragments were loaded onto an 8% polyacrylamide gel, which was made with a denaturing gradient ranging from 30 to 50%. The denaturant (100%) contained 7 M urea and 40% formamide. Gels were run at 130 V in 1 × TAE (40 mM Tris, 20 mM acetic acid, 1 mM EDTA, pH 8.0). After electrophoresis, gels were stained for 20 min in 5 μg/ml of ethidium bromide and de-stained for 10 min in 1 × TAE.

### Band excision from DGGE gels, re-amplification

DGGE gel bands were excised using a sterile scalpel, washed once in 1 × PCR buffer and incubated in the same buffer overnight at 4 °C; 5 μl of the buffer solution formed the template for PCR amplification. Re-amplification was conducted using the eubacterial PCR primers (with no ‘GC clamps’) depending on the primer set used in the DGGE, and with the same conditions as for the PCR preceding the DGGE analysis.

### Sequencing and sequence analysis

To verify the specificity of the PCR product, the DNA band from the polyacrylamide gel was cut out, and the purified DNA was extracted with Gel extraction kit (QIAGEN, Hilden, Germany) and sequenced using the BigDye Terminator Cycle Sequencing Kit (Applied Biosystems Inc., Foster City, USA) on an ABI PRISM™ 377 Genetic Analyzer (Applied Biosystems Inc., Foster City, USA). Analysis of the partial sequences was conducted using the Genbank DNA database and the BLAST algorithm. Identities of isolates were determined on the basis of the highest identity score.

### Statistical analysis

The statistical analysis of the obtained data concentrated on the one side on the descriptive statistics of the elemental concentrations in urinary stone samples analyzed with the TXRF method, the goodness of fit tests for element concentration distributions, and on the multigroup comparison of element concentrations and elemental correlation analysis with application of the type of urine stone as a grouping factor. The statistical methods and procedures, as well as detailed results of performed analysis are presented in our previous work (Kubala-Kukuś et al. 2017). Summarizing, in elemental analysis, 21 elements were determined, i.e., 9 uncensored (P, K, Ca, Fe, Zn, Ni, Br, Sr and Pb) and 12 censored elements (Mg, S, Cl, Ti, V, Cr, Mn, Cu, Se, Rb, I and Bi). The parameters of concentration distributions were calculated, in the case of censored element using survival approach and Kaplan–Meier estimator. XRPD measurements allowed specifying minerals present in the stones and, next, classifying the kidney stones.

On the other hand the main aim of the performed studies presented in this paper was to check the correlation of the crystalline and elemental composition of urinary stones with history of bacterial infections. In statistical analysis, the possible correlations were analyzed using Spearman’s correlation coefficient which is a nonparametric measure of statistical dependence between two variables (both for numerical and ordinal variables). In these studies, the correlations between crystalline substances identified in urinary stones were calculated, then the correlations between species of bacteria, followed by the correlations between the chemical composition of urinary stones and species of bacteria, and finally the correlations between the concentration of the elements and the species of bacteria. For each value of Spearman’s correlation coefficient, the statistical test was also performed verifying that it is statistically different from zero (statistically significant correlation). The correlation was interpreted as statistically significant for *p* value less than 0.05 (*p* < 0.05). All calculations and statistical analyses were performed using STATISTICA version 11.0 (StatSoft, Poland).

## Results

### DGGE analysis of bacterial DNA from urine stones using eubacterial-specific primers

Using universal eubacterial primers targeting the 16S rRNA, we identified several bacterial species present in the urine stones. Overall, different bacterial species were detected: *Escherichia coli* (fragment 14), *Klebsiella pneumoniae* (fragment 7), *Morganella morganii* (fragment 8, 12), *Proteus mirabilis* (fragment 1), *Proteus vulgaris* (fragment 2, 13), *Providencia rettgeri* (fragment 4, 9), *Providencia stuartii* (fragment 11), *Pseudomonas aeruginosa* (fragment 14), *Serratia marcescens* (fragment 5, 10), *Staphylococcus aureus* (fragment 6), *Streptococcus pyogenes* (fragment 3) (Online Resource1).

**Fig. 1:**
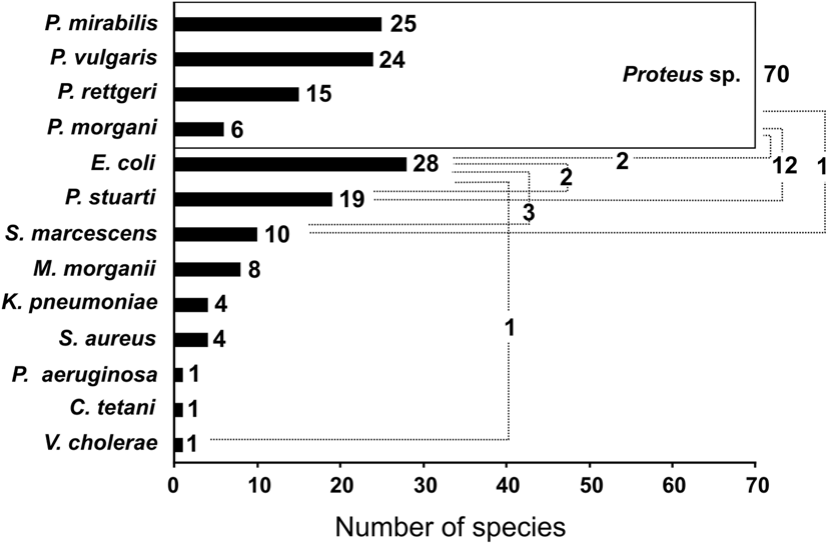
The number of identified bacterial species in 83 urinary stones using DGGE-PCR method. The number of identified bacterial species in alone and mixed infections in different types of stones (dashed line) are denoted

The homology between the sequence obtained from the DGGE bands and the closest species from the database are presented in Table 1. Figure 1 shows that *Proteus* sp. dominated in tested urine stones 84%, mainly *P. mirabilis* and *P. vulgaris*. In 17% of cases, *P. stuartii* was detected with *Proteus* spp. *E. coli* was the second most detected bacteria in urine stones (34%). The other species were identified on the level in range from 5 to 12%: *S. aureus*, *K. pneumoniae*, *M. morganii* and *S. marcescens*, respectively. *P. aeruginosa*, *C. tetani* and *V. cholerae* were identified only in single cases.

**Table 1:** BLAST analysis of urine stone–bacterial 16S rRNA sequences of excised fragments from DGGE gels

Species	Homology (%)
*Escherichia coli* ATCC 25922	92
*Klebsiella pneumoniae* ATCC 13883	87
*Morganella morganii* ATCC 25830	96
*Proteus mirabilis* ATCC 10005	92
*Proteus vulgaris* ATCC 6896	98
*Providencia rettgeri* ATCC 9250	97
*Providencia stuartii* ATCC 29914	91
*Pseudomonas aeruginosa* ATCC 25668	94
*Serratia marcescens* ATCC 14764	95
*Staphylococcus aureus* ATCC 25923	97
*Streptococcus pyogenes* ATCC 19615	94

### Crystalline and elemental composition of urinary stones: XRPD and TXRF techniques

**Fig. 2:**
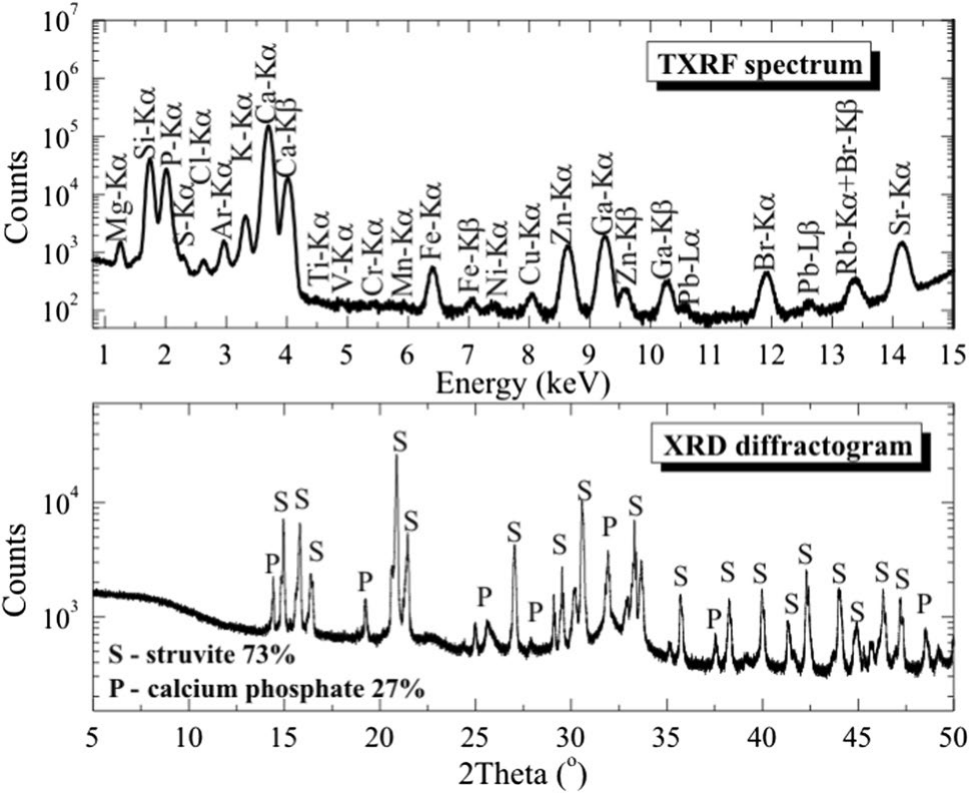
The upper panel: an example of the total reflection X-ray fluorescence (TXRF) spectrum of a human kidney stone sample excited by the primary X-rays generated in a Mo-anode X-ray tube operated with voltage *U* = 50 kV and current *I* = 600 µA. The measurement time was 30 min. The lower panel: a X-ray powder diffraction (XRPD) diffractogram of a human kidney stone sample. The primary X-rays were generated in a Cu-anode X-ray tube operated with voltage *U* = 45 kV and current *I* = 40 mA

**Fig. 3:**
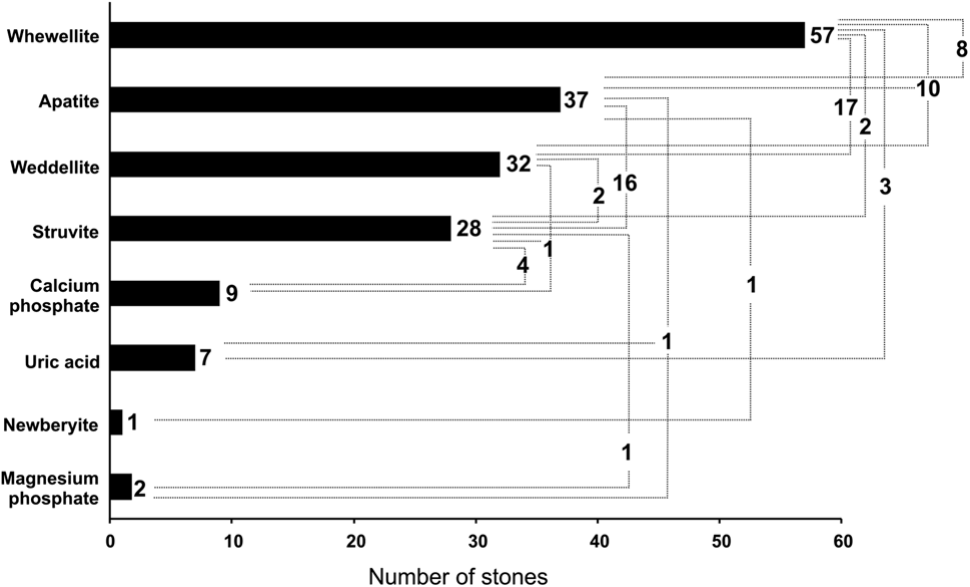
The number of urinary stones in which crystalline composition was determined using XRPD method. The number of urinary stones in which single or complex crystalline structures were detected (dashed line) are denoted

Figure 2 shows an example of the total reflection X-ray fluorescence (TXRF) spectrum and X-ray powder diffraction (XRPD) diffractogram of a human kidney stone sample. Figure 3 shows the crystalline composition of urinary stones. The stones contain several crystalline substances, namely, apatite (Ca_5_[(F,Cl,OH)(PO_4_)_3_]), struvite (NH_4_MgPO_4 _· 6H_2_O, uric acid (C_5_H_4_N_4_O_3_), weddellite (Ca(COO)_2 _· 2H_2_O), whewellite (Ca(COO)4_2 _· H_2_O), magnesium phosphate (Mg_3_(PO_4_)_2_) and calcium phosphate (Ca_3_(PO_4_)_2_). Eighty-three urinary stones examined by XPRD were divided into groups depending on their chemical composition: whewellite (24.1% alone; 20.5% with weddellite; 12% with weddellite and apatite; 9.6% with apatite; 2.4% with struvite), apatite (2.4% alone; 19.3% with struvite; 1.2% with uric acid stones and magnesium phosphate), weddellite (2.4% alone or with struvite; 1.2% with calcium phosphate and struvite), struvite (2.4% alone; 4.8% with calcium phosphate), calcium phosphate (4.8% alone), and uric acid (3.6% alone). Generally, the stones of mixed composition which contained minerals from different chemical groups dominated (79.5%), mainly whewellite with weddellite or apatite with struvite. The most frequently determinated is whewellite > apatite > weddellite > struvite in urinary stones. Detailed results of XRPD as well as TXRF analyses on elemental composition of 83 urinary stones used in this study are previously published (Kubala-Kukuś et al. 2017).

### Incidence analysis of the bacterial species versus the chemical and elemental composition of urinary stones

Table 2 shows the correlation between crystalline substances identified in urinary stones. It seems to be significant that two positive correlations of chemical compositions dominate in analyzed probes: weddellite with whewellite (0.268) or struvite with calcium phosphate (0.243). It is important that both chemical compositions are mutually exclusive: struvite with whewellite or weddellite (− 0.727 and − 0.303, respectively) and calcium phosphate with whewellite (− 0.349). The analyses of Spearman’s rank coefficients between species of bacteria identified in urinary stones (Table 3) show that *Proteus* species are correlated together (0.253–0.473) in contrast to *E. coli* (− 0.286 to − 0.335). Similarly, statistical analyses in this study shown that the presence of *E. coli* in urinary stones is mutually exclusive to the presence of *M. morganii* (− 0.233). *M. morganii* as previously classified *Proteus morganii*, belongs to the tribe of the family *Enterobacteriaceae* and it role in urinary stone formation is similar to *Proteus* spp. in contrast to *E. coli*. Table 4 shows that bacterial species are strongly positively correlated with chemical composition of urinary stones: *Proteus* spp. mainly with struvite (0.261–0.420) and newberyite (*P. rettgeri*; 0.235) but not correlated with weddellite and whewellite (− 0.221 to − 0.349). The presence of *E. coli* is characteristic for weddellite (0.272) and whewellite (0.207) but not for struvite (− 0.240). Moreover, *M. morganii* was identified in whewellite urinary stones (0.221) but does not contain struvite (− 0.233) or apatite (− 0.211). The analysis of elemental composition of urinary stones (Table 5) shows that the presence of *Proteus* spp. is correlated mainly with strontium (3 species; 0.256–295), phosphorus (3 species; 0.273–0.333), potassium (2 species; 0.328, 0.398), nickel (only *P. morgani*; 0.258) and zinc (only *P. mirabilis*; 0.284). *E. coli* in urinary stones is correlated only with iron (0.243) but not with potassium (− 0.225).

**Table 2:** Correlation between crystalline substances identified in urinary stones given by values of nonparametric Spearman’s rank correlation coefficients

	Uric acid	Apatite	Newberyite	Struvite	Weddellite	Whewellite	Calcium phosphate	Magnesium phosphate
Uric acid	–	− 0.185	− 0.034	− **0.217**	− **0.240**	− 0.075	0.034	− 0.034
Apatite	− 0.185	–	0.123	0.180	− 0.063	− 0.126	− 0.079	− 0.099
Newberyite	− 0.034	0.123	–	− 0.079	− 0.087	− 0.164	− 0.039	− 0.012
Struvite	− **0.217**	0.180	− 0.079	–	− **0.303**	− **0.727**	**0.243**	0.155
Weddellite	− **0.240**	− 0.063	− 0.087	− **0.303**	–	**0.268**	− 0.037	− 0.087
Whewellite	− 0.075	− 0.126	− 0.164	− **0.727**	**0.268**	–	− **0.349**	− 0.164
Calcium phosphate	0.034	− 0.079	− 0.039	**0.243**	− 0.037	− **0.349**	–	− 0.039
Magnesium phosphate	− 0.034	− 0.099	− 0.012	0.155	− 0.087	− 0.164	− 0.039	–

Statistically significant correlations are marked in bold (*p* value < 0.05)

**Table 3:** Correlation between species of bacteria given by values of nonparametric Spearman’s rank correlation coefficients

	*P. vulgaris*	*P. rettgeri*	*P. morgani*	*P. mirabilis*	*P. stuartii*	*E. coli*	*S. marcescens*	*P. aeruginosa*	*K. pneumoniae*	*M. morganii*	*S. aureus*	*C. tetani*	*V. cholerae*
*P. vulgaris*	–	**0.253**	0.130	**0.450**	0.159	**− 0.286**	**− 0.236**	**− **0.070	**− **0.144	**− **0.118	**− **0.144	**− **0.070	**− **0.070
*P. rettgeri*	**0.253**	–	**0.473**	0.169	0.117	**− 0.335**	**− **0.078	**− **0.052	**− **0.106	**− **0.153	**− **0.106	**− **0.052	**− **0.052
*P. morgani*	0.130	**0.473**	–	0.121	0.069	**− **0.199	**− **0.103	**− **0.031	**− **0.063	**− **0.091	**− **0.063	**− **0.031	**− **0.031
*P. mirabilis*	**0.450**	0.169	0.121	–	**− **0.045	**− 0.302**	**− **0.082	**− **0.073	**− **0.148	**− **0.036	**− **0.148	**− **0.073	**− **0.073
*P. stuarti*	0.159	0.117	0.069	**− **0.045	–	**− **0.207	**− **0.114	**− **0.060	0.011	**− **0.178	**− **0.123	**− **0.060	**− **0.060
*E. coli*	**− 0.286**	**− 0.335**	**− **0.199	**− 0.302**	**− **0.207	–	0.127	**− **0.079	**− **0.161	**− 0.233**	**− **0.042	**− **0.079	0.155
*S. marcescens*	**− 0.236**	**− **0.078	**− **0.103	**− **0.082	**− **0.114	0.127	–	**− **0.041	**− **0.083	**− **0.121	**− **0.083	**− **0.041	**− **0.041
*P. aeruginosa*	**− **0.070	**− **0.052	**− **0.031	**− **0.073	**− **0.060	**− **0.079	**− **0.041	–	**− **0.025	**− **0.036	**− **0.025	**− **0.012	**− **0.012
*K. pneumoniae*	**− **0.144	**− **0.106	**− **0.063	**− **0.148	0.011	**− **0.161	**− **0.083	**− **0.025	–	**− **0.073	**− **0.051	**− **0.025	**− **0.025
*M. morganii*	**− **0.118	**− **0.153	**− **0.091	**− **0.036	**− **0.178	**− 0.233**	**− **0.121	**− **0.036	**− **0.073	–	**− **0.073	**− **0.036	**− **0.036
*S. aureus*	**− **0.144	**− **0.106	**− **0.063	**− **0.148	**− **0.123	**− **0.042	**− **0.083	**− **0.025	**− **0.051	**− **0.073	–	**− **0.025	**− **0.025
*C. tetani*	**− **0.070	**− **0.052	**− **0.031	**− **0.073	**− **0.060	**− **0.079	**− **0.041	**− **0.012	**− **0.025	**− **0.036	**− **0.025	–	**− **0.012
*V. cholerae*	**− **0.070	**− **0.052	**− **0.031	**− **0.073	**− **0.060	0.155	**− **0.041	**− **0.012	**− **0.025	**− **0.036	**− **0.025	**− **0.012	–

Statistically significant correlations are marked in bold (*p* value < 0.05)

**Table 4:** Correlation between chemical composition of urinary stones and species of bacteria given by values of nonparametric Spearman’s rank correlation coefficients calculated for species of bacteria

Species of bacteria	Type of stone							
	Uric acid	Apatite	Newberyite	Struvite	Weddellite	Whewellite	Calcium phosphate	Magnesium phosphate
*P. vulgaris*	**− **0.194	0.123	**− **0.070	**0.332**	**− **0.068	**− 0.314**	0.034	**− **0.070
*P. rettgeri*	**− **0.143	0.146	**0.235**	**0.261**	**− **0.115	**− 0.290**	0.038	**− **0.052
*P. morgani*	**− **0.085	0.030	**− **0.031	0.194	**− 0.221**	**− **0.112	0.052	**− **0.031
*P. mirabilis*	**− **0.199	0.151	**− **0.073	**0.420**	**− **0.034	**− 0.349**	0.193	0.168
*P. stuarti*	**0.247**	0.031	**− **0.060	0.036	**− **0.137	**− **0.188	**− **0.006	**− **0.060
*E. coli*	**− **0.033	0.027	**− **0.079	**− 0.240**	**0.272**	**0.207**	**− **0.003	**− **0.079
*S. marcescens*	0.154	**− **0.034	**− **0.041	**− **0.186	0.087	0.170	**− **0.129	**− **0.041
*P. aeruginosa*	**0.364**	**− **0.099	**− **0.012	**− **0.079	**− **0.087	0.075	**− **0.039	**− **0.012
*K. pneumoniae*	0.134	0.025	**− **0.025	**− **0.042	0.053	0.031	**− **0.078	**− **0.025
*M. morganii*	0.048	**− 0.211**	**− **0.036	**− 0.233**	0.077	**0.221**	0.017	**− **0.036
*S. aureus*	**− **0.068	0.138	**− **0.025	**− **0.161	**− **0.063	0.152	**− **0.078	**− **0.025
*C. tetani*	**− **0.034	0.123	**− **0.012	0.155	**− **0.087	0.075	**− **0.039	**− **0.012
*V. cholerae*	**− **0.034	**− **0.099	**− **0.012	**− **0.079	**− **0.087	0.075	**− **0.039	**− **0.012

Statistically significant correlations are marked in bold (*p* value < 0.05)

**Table 5:** Correlation between element concentrations and species of bacteria given by values of nonparametric Spearman’s rank correlation coefficients

Species of bacteria	Element								
	P	K	Ca	Fe	Ni	Zn	Br	Sr	Pb
*P. vulgaris*	**0.311**	**0.328**	**− **0.112	**− **0.162	**− **0.114	0.187	0.048	0.180	0.091
*P. rettgeri*	**0.273**	0.165	0.034	**− 0.302**	0.029	0.146	0.001	**0.256**	0.067
*P. morgani*	0.206	0.198	0.101	**− **0.153	**0.258**	0.165	**− **0.033	**0.295**	0.019
*P. mirabilis*	**0.333**	**0.398**	**− **0.102	0.116	**− **0.094	**0.284**	**− **0.082	**0.270**	0.047
*P. stuarti*	**− **0.072	0.038	**− **0.213	**− **0.029	**− **0.084	**− **0.134	**− **0.026	**− **0.126	**− **0.134
*E. coli*	**− **0.187	**− 0.225**	0.103	**0.243**	0.056	**− **0.096	0.088	**− **0.114	0.149
*S. marcescens*	**− **0.188	**− **0.107	**− **0.051	0.002	0.057	**− **0.154	0.008	**− **0.168	**− **0.110
*P. aeruginosa*	**− **0.166	**− **0.023	**− **0.175	**− **0.161	**− **0.184	**− **0.157	0.092	**− **0.175	**− **0.175
*K. pneumoniae*	**− **0.049	**− **0.066	0.021	**− **0.073	0.077	0.073	**− **0.204	**− **0.023	**− **0.059
*M. morganii*	**− **0.196	**− **0.070	0.147	0.140	0.061	**− **0.184	0.063	**− **0.089	0.000
*S. aureus*	**− **0.054	**− **0.197	0.002	**− **0.195	0.131	0.085	0.042	0.028	0.127
*C. tetani*	0.069	0.005	0.120	**− **0.088	0.161	0.171	0.051	0.083	**− **0.018
*V. cholerae*	**− **0.111	**− **0.115	**− **0.074	0.101	**− **0.065	**− **0.138	**− **0.041	**− **0.101	0.115

Statistically significant correlations are marked in bold (*p* value < 0.05)

## Discussion

In this study, we identified DNA of bacterial uropathogens in 83 urinary stones using PCR and DGGE methods and correlated with their crystalline and elemental composition. Crystalline material is the primary constituent of most human urinary tract stones. Additionally, the urinary stones contain macromolecules, and other cellular and elemental components, termed the matrix, and reflect the urine chemistry and abnormalities in urinary tract physiology. These physico-chemical conditions have effects on the process of stone development and might be caused by bacterial infections. Although stones with mixed composition in different configurations dominate (79.5%), their statistical correlations with a history of infections show two general scenarios of particular “chemico-bacterial” compositions.

The first one is associated with *Proteus* spp. which dominated in 84% of infectious urinary stones measured in this study and is strongly correlated with struvite and calcium phosphate, in whose matrix additionally strontium, phosphorus, potassium, nickel and zinc are detected. Urinary tract infection by *Proteus* spp. occurs in patients requiring long-term maintenance of catheters in the urinary tract, and this type of infection coexists with nephrolithiasis and bladder lithiasis (Burall et al. 2004). The chemical composition of stones in the presence of *Proteus* spp. is well-known and this study confirms that the mechanism of struvite mineralization is associated with urease activity. The presence of urease-positive *Proteus* spp. in urine causes the elevation of the concentration of ammonia and carbon dioxide. Ammonia creates ammonium ion and an increase in pH of urine takes place. Carbon dioxide reacts with water creating the carbonic acid. Additionally, the formation of phosphate ions in urine is observed. These above chemical reactions lead to the supersaturation and crystallization of magnesium and calcium salts as struvite (MgNH_4_PO_4 _· 6H_2_O) and carbonate apatite (Ca_10_(PO_4_)_6 _· CO_3_), respectively (Rodman 1999; Torzewska and Rozalski 2014). Urease has a nickel-containing active site (Mobley and Island 1995). Moreover, zinc uptake by *P. mirabilis* provides a competitive advantage to other pathogenic bacteria, including uropathogenic *E. coli* (Sabri et al. 2009). This metal is required for virulence factors regulations, such as flagella and ZapA metalloprotease. Bacteria use flagella to swim through liquids and toward chemical gradients. This motility (swarming) plays a crucial role in *P. mirabilis* virulence and stimulates the expression of the other virulence factors, including urease and ZapA protease. Regulation of flagella is mediated through the class 1 flagellar master regulator genes *flhDC*, as FlhD_4_C_2_ protein, possessing zinc binding site. It indicates that zinc plays an important role in flagella expression. Moreover, the presence of functional zinc uptake system (*znuACB*) by *P. mirabilis* confirms importance of zinc for bacteria metabolism (Belas et al. 2004; Schaffer and Melanie 2015). Strontium has similar physico-chemical properties as calcium, and its absorption and excretion follow that of calcium (Vezzoli et al. 1998).

The second scenario for urinary stone mineralization presented in this study is associated with *E. coli* identified in mainly weddellite stones, in which iron was detected. Generally, published studies indicate that *E. coli* is a cause of urinary tract infections in about 80% of patients (Ronald 2003; Ramchandani et al. 2017). It seems to be more likely, that *E. coli*-mediated scenario is more often observed due to the frequency of this bacterial infection. However, mainly *Proteus* spp. may serve as centers for heterogeneous nucleation and urinary stones growth and our work confirms clinical studies which indicate urease activity as crucial in such urinary stone formation. The results of genetic and biophysical studies presented in this work showed that the first scenario of *Proteus* stimulated urinary stone formation that is dominating from the clinical point of view.

Barr-Beare et al. showed that *E. coli* was identified in patients by calcium oxalate stone culture. *E. coli* selectively aggregated on and around calcium oxalate monohydrate crystals (whewellite) in in vitro studies (Barr-Beare et al. 2015). Our study shows that *E. coli* is statistically significantly correlated with whewellite and weddellite stones. Low availability of iron is a major limiter of bacterial growth during host colonization, so many pathogenic bacteria, such as UPEC, evolved specific iron-transport systems and number of chelating compounds (siderophores). Iron is a cofactor of many bacterial proteins, which are involved in electron transport, reactive oxygen species detoxification, amino acid or nucleoside synthesis.

Interestingly, the aforementioned scenarios of urinary stones formation mediated by *Proteus* spp. and *E. coli* are totally independent. The statistical correlations of bacterial infections with crystalline and elemental composition of bladder stones showed that in mixed bacterial infections, one scenario dominated and excluded the second one. In our opinion, this is the most important and new observation presented in this study.

Additionally, we observed in this study that *P. stuartii* was identified in various types of analyzed stones, which is surprising because this pathogen corresponds to only 3% of urinary tract infections described in literature. The biophysical analyses in this study confirm that *P. stuartii* as an uropathogen with similar biochemical activity mainly to *Proteus* spp., might play an important role in urinary stone formation (O’Hara et al. 2000). *P. aeruginosa* is a cause of urinary tract infection in 4.5–7.6% (Akkoyun et al. 2008; Piljić et al. 2009). In our study, *Pseudomonas aeruginosa* was identified only in the uric and mixed stones. *K. pneumoniae* may be the cause of urinary tract infections from 8.5 to 10% (Johansen et al. 2006). We found it in all kinds of stones, from 2.7% in infected phosphate stones to 14.3% in the stones of uric acid. *M. morganii* was found in the urate, oxalic and mixed stones, but it has not been identified in phosphate ones. *M. morganii* may cause bacteriuria in 37% of patients with urinary tract infections (Lee and Liu 2006).

## Electronic supplementary material

Below is the link to the electronic supplementary material.

Supplementary material 1 (DOCX 101 kb)
