# Supplementary material for: The correlation of crystalline and elemental composition of urinary stones with a history of bacterial infections: TXRF, XRPD and PCR-DGGE studies
Source: Eur Biophys J. 2018 Nov 27;48(1):111–8. doi: 10.1007/s00249-018-1338-7 (PMC6330562; doi:10.1007/s00249-018-1338-7)
Supplement: Supplementary file 1 — Supplementary material 1 (DOCX 101 kb) [file 249_2018_1338_MOESM1_ESM.docx]

**SUPPLEMENTARY MATERIAL**

**Article title**:

The correlation of crystalline and elemental composition of urinary stones with a history of bacterial infections – TXRF, XRPD and PCR-DGGE studies

**Journal name**:

# European Biophysics Journal with Biophysics Letters

**Author names**:

Michał Arabski , Ilona Stabrawa, Aldona Kubala-Kukuś, Katarzyna Gałczyńska, Dariusz Banaś, Łukasz Piskorz, Ewa Forma, Magdalena Bryś, Waldemar Różański, Marek Lipiński

corresponding author: phone/fax: +48 41 349 63 31; e-mail: arabski@ujk.edu.pl (MA).

**Affiliation and e-mail address of the corresponding author**:

Department of Biochemistry and Genetics, Institute of Biology, Jan Kochanowski University, Świętokrzyska St. 15, 25-406, Kielce, Poland, e-mail: arabski@ujk.edu.pl


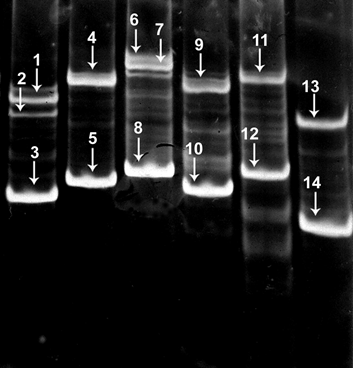


**Online Resource1** DGGE profiles of the total urine stones bacteria species. Lane 1- *Proteus mirabilis,* lane 2, 13 - *Proteus vulgaris*[,](http://www.google.pl/url?sa=t&source=web&cd=4&ved=0CDMQFjAD&url=http%3A%2F%2Fwww.pol-aura.pl%2Fproteus-vulgaris-atcc-6896-p-39106.html&ei=PjcbTo_RFcWhOviW5PUI&usg=AFQjCNHq8JjWTZ7mfMfHXNGlVMw84ktA6A) lane 3 *- Streptococcus pyogenes,* lane 4, 9 *- Providencia rettgeri,* lane 5, 10 *- Serratia marcescens,* lane 6 *- Staphylococcus aureus,* lane 7 *- Klebsiella pneumoniae,* lane 8, 12 *- Morganella morganii,* lane 11 *- Providencia stuartii,* lane 14 *- Escherichia coli.*
